# Early Predictors of Outcome in Pediatric Acquired Demyelinating Syndromes: A Retrospective Study Stratified by Final Diagnosis

**DOI:** 10.3390/children12080975

**Published:** 2025-07-24

**Authors:** Emanuela Claudia Turco, Martina Gnazzo, Sara Giordani, Giulia Pisanò, Valentina Baldini, Elena Giroldini, Benedetta Piccolo, Cosimo Neglia, Susanna Esposito, Maria Carmela Pera

**Affiliations:** 1 Child Neuropsychiatry Unit, Department of Medicine and Surgery, University of Parma, 43124 Parma, Italy; eturco@ao.pr.it (E.C.T.); sara.giordani@studenti.unipr.it (S.G.); elenagiroldini@gmail.com (E.G.); bpiccolo@ao.pr.it (B.P.); mariacarmela.pera@unipr.it (M.C.P.); 2 Department of Biomedical, Metabolic and Neural Sciences, University of Modena and Reggio Emilia, 41121 Emilia-Romagna, Italy; martignazzo@hotmail.it (M.G.); giuliapisan@gmail.com (G.P.); 3 Department of Biomedical and Neuromotor Sciences, University of Bologna, 40126 Bologna, Italy; 4 Pediatric Clinic, University Hospital, Department of Medicine and Surgery, University of Parma, 43124 Parma, Italy; negliamino@gmail.com (C.N.); susannamariaroberta.esposito@unipr.it (S.E.)

**Keywords:** pediatric demyelinating syndromes, multiple sclerosis, MOGAD, outcome prediction, disease-modifying therapy, evoked potentials, magnetic resonance imaging, EDSS, neuroimmunology, childhood CNS disorders

## Abstract

**Background/Objectives**: Pediatric acquired demyelinating syndromes (ADSs) encompass a heterogeneous group of disorders, including multiple sclerosis (MS), MOG antibody-associated disease (MOGAD), and neuromyelitis optica spectrum disorder (NMOSD), with distinct clinical trajectories and prognoses. While analyzed collectively at baseline to reflect real-world diagnostic uncertainty, outcome predictors were also examined according to final diagnosis. Identifying early predictors is crucial for optimizing long-term outcomes. **Methods**: We retrospectively analyzed 30 pediatric patients (mean onset age: 11.3 years) with ADSs. Clinical, radiological, CSF, antibody, and neurophysiological data were collected and analyzed alongside treatment strategies. Outcomes—EDSS scores, neuroradiological changes, and clinical status—were evaluated over a 3-year period. **Results**: Final diagnoses included MOGAD (36.6%), MS (33.3%), NMOSD (6.6%), ADEM (10%), and other ADSs (13.3%). At onset, ≥3 brain lesions were present in 76.7% of patients. Disease-modifying therapies (DMTs) were used in 37% and acute immunotherapy in 90%. EDSS progression was significantly associated with DMT use at multiple timepoints, with additional predictors including MRI lesion type, CSF findings, antibody status, and evoked potentials. At 3 years, neurocognitive function predicted clinical outcome. **Conclusions**: Early immunotherapy and baseline instrumental findings are key predictors of outcome in pediatric ADSs. MOGAD showed a more favorable course, while MS and NMOSD were associated with greater long-term disability. A comprehensive, early diagnostic approach is essential for improving prognosis.

## 1. Introduction

Acquired demyelinating syndromes (ADSs) of the central nervous system (CNS) in children encompass a spectrum of immune-mediated disorders, including multiple sclerosis (MS), myelin oligodendrocyte glycoprotein antibody-associated disease (MOGAD), and aquaporin-4 antibody-positive neuromyelitis optica spectrum disorder (AQP4-NMOSD). These conditions, though rare in the pediatric population, may lead to significant neurological disability over time through both inflammatory and degenerative mechanisms [1,2].

Clinical presentation is highly variable depending on the affected CNS regions—optic nerves, spinal cord, or brain parenchyma—and ranges from isolated visual loss or motor symptoms to multifocal encephalopathy. Early in the disease course, differential diagnosis is often challenging, particularly when distinguishing monophasic episodes (such as acute disseminated encephalomyelitis or isolated optic neuritis) from chronic relapsing conditions like MS or AQP4-NMOSD [3].

Epidemiological data estimate a global annual incidence of pediatric ADS at approximately 0.87 per 100,000 children. MS accounts for nearly 20% of these cases, although pediatric-onset MS represents fewer than 5% of all MS diagnoses [4]. MOG antibodies are detected in around 30% of pediatric ADS cases, particularly in children under eleven years of age, whereas AQP4-NMOSD remains relatively uncommon [5]. In a North American study of 267 pediatric ADS patients, 71% exhibited a monophasic course; among these, 61% were MOG-Ab-positive at onset, and relapses were rare in children who did not satisfy diagnostic criteria for MS or AQP4-NMOSD [6,7].

Prompt identification of children at higher risk of relapse or disability progression is essential to guide early initiation of disease-modifying therapy and optimize monitoring strategies. However, prognostic markers validated in pediatric ADSs remain limited.

This study aimed to characterize the clinical, laboratory, and MRI features of pediatric ADS patients at a single tertiary center and to explore potential predictors of disease evolution, relapse risk, and disability progression, particularly clinical outcome and longitudinal disability, as measured by the Expanded Disability Status Scale (EDSS).

## 2. Materials and Methods

### 2.1. Participants and Inclusion Criteria

This study included 30 children and adolescents, aged 2 to 17 years, who experienced the onset of demyelinating disease between March 2013 and November 2024. All participants underwent diagnostic procedures and neurological follow-up at the Child Neuropsychiatry Unit of Parma Hospital.

The patients were selected according to the 2013 International Pediatric Multiple Sclerosis Study Group (IPMSSG) criteria (which refers to the 2010 McDonald criteria for neuroradiological dissemination in time (DIT) and space (DIS)), the international consensus diagnostic criteria for neuromyelitis optica spectrum disorders of 2015, and the International MOGAD Panel proposed criteria of 2023.

Although initial analyses considered the entire cohort, stratified evaluations were performed according to final diagnoses (MS, MOGAD, NMOSD, ADEM, and others) to account for the known clinical and biological differences across these conditions. The rationale for analyzing the cohort collectively lies in the shared early diagnostic pathway and the importance of identifying predictors that may be present even before a definitive nosological classification.

Patients with other concomitant neurological conditions were excluded from the study.

Furthermore, patients diagnosed with autoimmune encephalitis (e.g., anti-NMDA receptor encephalitis) without radiological or clinical features compatible with acquired demyelinating syndromes were excluded, in accordance with current diagnostic consensus definitions.

The subgroup labeled as “other demyelinating disorders” (13.3%) included two patients with isolated optic neuritis and two with isolated transverse myelitis, all of whom had negative MOG and AQP4 antibodies and did not develop relapsing disease or fulfill diagnostic criteria for MS, MOGAD, NMOSD, or ADEM during follow-up.

The study was approved by CET (Comitato Etico Area Vasta Emilia Nord) and authorized by the General Director of Parma Maggiore Hospital (protocol no. 0002714).

### 2.2. Data Collection

The data utilized in this study were collected through the review of outpatient and inpatient medical records, as well as the analysis of instrumental and laboratory test results. Finally, the outcomes of neuropsychological tests performed on patients were extracted from the neuropsychology archive of the Child Neuropsychiatry department.

The data were collected at baseline (T0), corresponding to disease onset, after 1 month and 6 months for the short-term follow-up, and subsequently on an annual basis for the long-term follow-up until its conclusion. The end of the follow-up coincided with either the patient’s discharge or the most recent follow-up available.

The selected patients were comprehensively described by creating a baseline profile at the time of the initial attack, taking into account anamnestic characteristics, mode of onset, brain and spine MRI findings, cerebrospinal fluid (CSF) analysis, neurocognitive impairment and level of disability (assessed using the EDSS score), electroencephalogram results, and evoked potentials testing.

At each follow-up visit, neuroimaging findings, clinical progression, EDSS score, and response to disease-modifying drugs (if administered) were evaluated.

### 2.3. Statistical Analysis

All statistical analyses were performed using the JASP v.12.2 software. Descriptive statistics were computed for all demographic, clinical, and instrumental variables. Continuous variables were reported as means and standard deviations (SD), while categorical variables were reported as frequencies and percentages. Group comparisons were conducted using the Chi-square test or Fisher’s exact test for categorical variables and one-way ANOVA or Kruskal–Wallis tests for continuous variables, as appropriate. Associations between predictors and outcomes (both clinical and radiological) were evaluated using univariate analyses, followed by multivariate logistic regression. Correlation analyses were conducted using Pearson’s correlation coefficient. A *p*-value < 0.05 was considered statistically significant.

## 3. Results

A total of 30 pediatric patients (50% male) with acquired demyelinating syndromes were included in the study. The mean age at disease onset was 11.3 years (range: 2–17), with 50% of patients presenting before the age of 11. A positive family history for neurological disorders—such as stroke, epilepsy, Parkinson’s disease, or oligodendroglioma—was reported in 13% of cases. Additionally, a family history of autoimmune diseases was found in 20% of patients, including conditions such as autoimmune cholangitis, rheumatoid arthritis, psoriasis, Hashimoto’s thyroiditis, vitiligo, and celiac disease. Epstein–Barr Virus (EBV) exposure was documented in 53% of patients. Cerebrospinal fluid (CSF) analysis revealed hyperproteinorrachia in 33% of the sample.

Regarding final diagnoses, 33.3% of patients were diagnosed with multiple sclerosis (MS), 36.6% with myelin oligodendrocyte glycoprotein antibody-associated disease (MOGAD), 6.6% with neuromyelitis optica (NMO), 10% with acute disseminated encephalomyelitis (ADEM), and 13.3% with other demyelinating disorders.

The “other demyelinating disorders” group (13.3%) consisted of four patients: two with isolated optic neuritis and two with isolated transverse myelitis. All of these cases were negative for MOG and AQP4 antibodies and did not evolve into MS, MOGAD, NMOSD, or ADEM over the follow-up period, thus remaining classified as monophasic demyelinating events (Table 1).

At disease onset, motor disturbances and altered deep tendon reflexes were reported in 20 patients (66.7%), while sensory disturbances, limb or back pain, and autonomic symptoms were present in 15 patients (50.0%). Visual symptoms were noted in 14 patients (46.7%), and disorders of consciousness, meningeal signs (e.g., Brudzinski’s sign, nuchal rigidity), headache, and behavioral changes were reported in 11 patients (36.7%). Coordination and balance issues were also observed in 11 patients (36.7%), cranial nerve involvement in 3 patients (10.0%), and seizures in 1 patient (3.3%).

Cerebrospinal fluid (CSF) analysis showed elevated protein levels (hyperproteinorrachia) in 33.3% and pleocytosis in 56.7% of patients. Oligoclonal band analysis revealed the following: 30.0% met pattern 1 (no bands in serum or CSF), 33.3% met pattern 2 (intrathecal IgG synthesis), 10.0% showed pattern 3 (combined intrathecal and shared serum–CSF bands), and 26.7% showed pattern 4 (identical bands in serum and CSF, indicating systemic synthesis).

All patients underwent brain MRI. The number of lesions was ≥3 in 76.7% and <3 in 23.3%. Lesion patterns were categorized as circumscribed (40.0%), diffuse (46.7%), or optic-neuritis-associated (13.3%). Regarding lesion location, 16.7% had spinal/supratentorial lesions, 33.3% had supra- and infratentorial lesions, 36.7% had supra-/infratentorial and spinal lesions, and 13.3% had isolated spinal lesions. Spinal cord involvement, either isolated or associated with brain lesions, was observed in 66.7% (20/30) of patients.

Evoked potentials were conducted in 12 patients and revealed pathological findings in 20% of the total cohort.

Therapeutic management at clinical onset was analyzed. Of the 30 patients, 27 received acute-phase treatment, while 3 patients (2 with MS and 1 with MOGAD) had spontaneous resolution without pharmacological intervention. Among the treated subgroup, 29.6% received intravenous methylprednisolone alone, while others received combination treatments: IVMP plus oral tapering (37.0%), IVMP plus IVIG (14.8%), IVMP plus IVIG and tapering (11.1%), IVIG plus PLEX (3.7%), and IVMP plus PLEX (3.7%).

Disease-modifying treatment was initiated in 11 patients: 27.3% received Natalizumab, 27.3% received Fingolimod, and 45.5% received Rituximab.

While Natalizumab and Fingolimod were prescribed exclusively for MS, Rituximab was used in NMOSD cases, reflecting current off-label practice in pediatric neuroimmunology.

### Predictors of Clinical and Radiological Outcome

For the entire cohort (N = 30), clinical follow-up data were available at 1 month, for 28 patients at 6 months, for 24 patients at 1 year, for 19 patients at 2 years, for 14 patients at 3 years, and for 9 patients at 4 years. Due to missing data, the follow-up analysis was limited to the first four years from disease onset, while statistical analyses of the same variables were conducted up to 3 years. Specifically, clinical evolution, neuroradiological findings, and Expanded Disability Status Scale (EDSS) scores were monitored over time.

**Figure 1:**
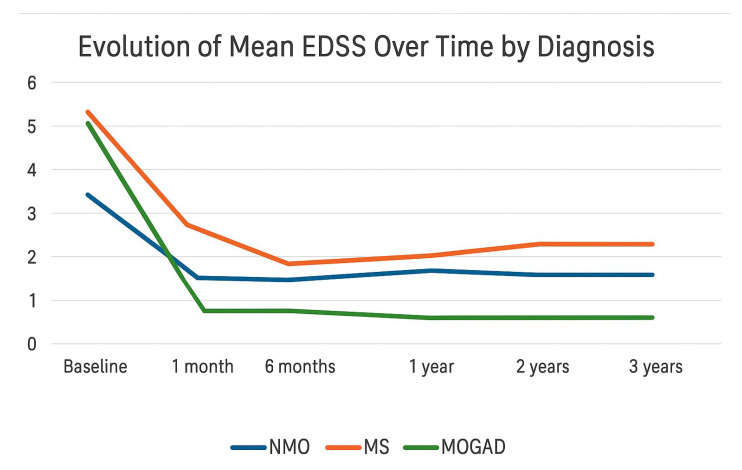
Evolution of mean EDSS over time by diagnosis.

The trend of mean EDSS scores was analyzed according to diagnosis: NMOSD, MS, and MOGAD. Multiple sclerosis (MS) showed the worst progression in terms of disability, with a higher average EDSS score compared to NMOSD and MOGAD from the first month after onset, despite a similar baseline score across the three conditions. After an initial decrease within the first six months, the EDSS score progressively increased starting from the first year, indicating a gradual accumulation of disability over time, consistent with the chronic and potentially progressive nature of the disease. In contrast, MOGAD demonstrated a significantly more favorable trajectory: the EDSS score declined rapidly within the first month and remained consistently low, close to zero, throughout the three-year follow-up, suggesting a more complete and sustained clinical recovery. NMOSD occupied an intermediate position, with a high EDSS score at onset that stabilized over time at levels lower than those seen in MS but without reaching the degree of recovery observed in MOGAD. However, data on NMOSD are limited by the small number of patients included in the cohort (only two cases), which warrants caution in interpreting the results (Figure 1).

A statistical significance analysis (*p* < 0.05) was conducted to identify predictive factors over time.

For EDSS progression, at 1 month, the only variable significantly associated with outcome was the presence of abnormal evoked potentials. At 6 months, significant predictors included lesion type on MRI, CSF oligoclonal band pattern, and DMT. At 1 year, significant variables were evoked potentials, lesion type on MRI, CSF pattern, diagnosis, presence of serum antibodies, use of DMT, and DMT type.

**Figure 2:**
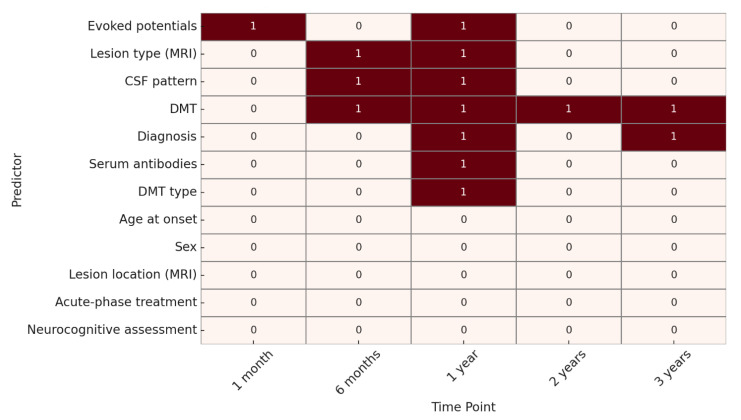
Heatmap of predictive factor significance for EDSS evolution. **Legend:** 🟥 **red squares (1)** = predictor significantly associated with the outcome (*p* < 0.05); ⬜ **white squares (0)** = no statistically significant association.

At 2 years, DMT remained the only statistically significant predictor, while at 3 years, both diagnosis and DMT were significantly associated with EDSS outcome. (Figure 2).

**Figure 3:**
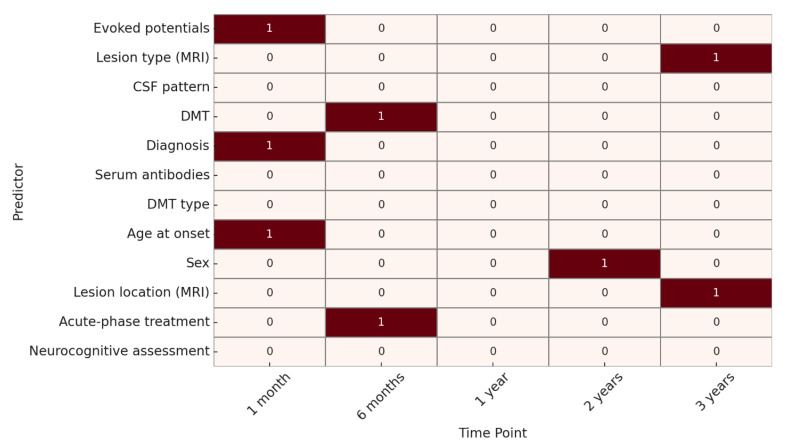
Heatmap of predictive factor significance for neuroradiological evolution. **Legend:** 🟥 **red squares (1) = predictor significantly associated with the outcome (*p* < 0.05)**; ⬜ white squares (0) = no statistically significant association.

Regarding neuroradiological progression, at 1 month, significant predictors were age at onset, evoked potentials, and specific diagnosis, when already defined through specific biomarkers, such as MOG or AQP4 antibodies. At 6 months, acute-phase treatment and DMT were significant. At 1 year, no factors reached statistical significance. At 2 years, only sex was significantly associated, while at 3 years, lesion type and lesion location on MRI were identified as significant predictors. (Figure 3).

As for clinical outcome, at 1 month, significant predictors included sex, lesion location on MRI, serum antibodies, and diagnosis. At 6 months, 1 year, and 2 years, no variables showed statistically significant associations.

**Figure 4:**
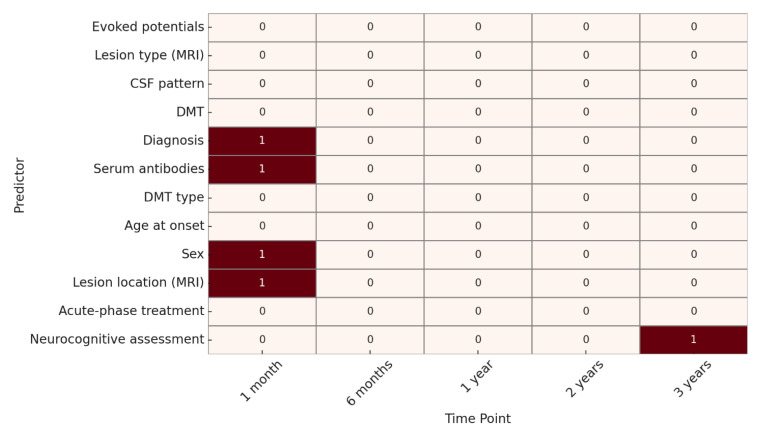
Heatmap of predictive factor significance for clinical evolution. **Legend: 🟥 red squares (1) = predictor significantly associated with the outcome (*p* < 0.05)**; ⬜ **white squares (0)** = no statistically significant association.

At 3 years, only the neurocognitive assessment was significantly associated with clinical evolution. (Figure 4).

**Figure 5:**
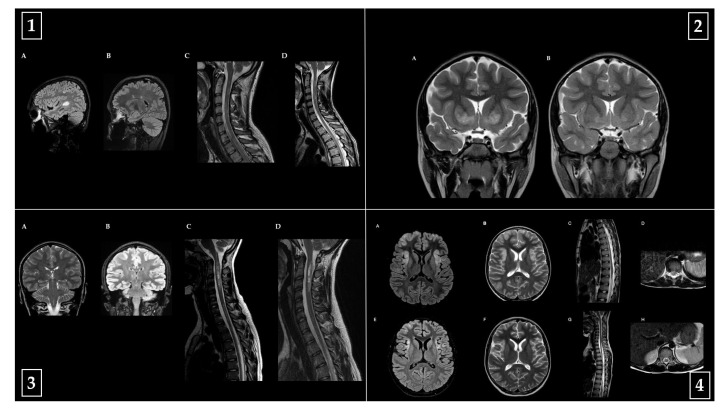
Representative brain and spinal cord MRI findings from four pediatric patients with distinct acquired demyelinating disorders of the central nervous system. All images include acute-phase and follow-up studies. **(1) Multiple sclerosis (MS):** MRI of an 18-year-old male patient with a diagnosis of MS. (**A**,**C**) Acute phase: sagittal FLAIR brain MRI (**A**) demonstrates multiple hyperintense lesions in supratentorial and infratentorial regions, including the left temporal peritrigonal white matter and brainstem, with incomplete ring enhancement of the temporal lesion. Sagittal T2-weighted cervical spinal MRI (**C**) reveals extensive intramedullary hyperintensities from C2 to C6, predominantly involving the posterior columns, with marginal ring enhancement at C4–C5. A separate lesion is noted in the left lateral cord at D10–D11. (**B**,**D**) Follow-up imaging: brain MRI (**B**) shows stable lesion burden without new enhancement, while cervicodorsal MRI (**D**) confirms persistence of spinal lesions without new findings. **(2) Acute disseminated encephalomyelitis (ADEM):** MRI of a 5-year-old female patient with a diagnosis of ADEM. (**A**) Coronal T2-weighted brain MRI during the acute phase shows bilateral and symmetrical hyperintense lesions involving the basal ganglia (caudate heads and anterior pallido-putaminal regions), tegmentum, periaqueductal and hypothalamic periventricular gray matter, and optic tracts. Moderate enlargement of the convexity subarachnoid spaces and thinning of the mid-posterior corpus callosum are also evident. (**B**) At follow-up, a faint residual hyperintensity persists in the globi pallidi, with reduced ventricular and subarachnoid space enlargement. No contrast enhancement is observed at either timepoint. **(3) Neuromyelitis optica spectrum disorder (NMOSD):** MRI of a 16-year-old female patient with a diagnosis of NMOSD. (**A**,**C**) Acute phase: coronal T2-weighted brain MRI (**A**) shows a faint lesion in the dorsal medulla near the area postrema and a focal lesion in the right ventro-thalamic region. Sagittal T2-weighted spinal MRI (**C**) reveals a longitudinally extensive lesion from the cervicomedullary junction to C4, with cord swelling, bright spotty lesions, and intense posterior nodular enhancement. A thoracic lesion is visible at D5–D6, with subpial serpiginous enhancement. (**B**,**D**) Follow-up imaging: brain MRI (**B**) shows persistence of the right thalamic lesion without new abnormalities; spinal MRI (**D**) reveals reduced lesion length and residual pseudocystic and nodular components, particularly in the posterior cord at C2–C4 and dorsolateral thoracic cord at D5–D6. **(4) MOG antibody-associated disease (MOGAD):** MRI of a 13-year-old male patient with a diagnosis of MOGAD. (**A**–**D**) Acute phase: axial FLAIR (**A**) and T2-weighted (**B**) brain MRI demonstrate bilateral multifocal lesions in the cortical and subcortical regions of the temporal, insular, and frontal lobes. Sagittal (**C**) and axial (**D**) T2-weighted spinal MRI shows a linear hyperintense lesion affecting the conus medullaris. (**E**–**H**) Follow-up imaging shows partial resolution of cerebral lesions on FLAIR (**E**) and T2 (**F**) and complete resolution of spinal abnormalities in both sagittal (**G**) and axial (**H**) views.

Representative baseline and follow-up MRI images from one patient per diagnostic category (MS, MOGAD, NMOSD, ADEM) are shown in Figure 5, highlighting key radiological features and their evolution over time.

All patients with ADEM had a monophasic disease course, with no clinical relapses or new radiological lesions during follow-up.

## 4. Discussion

This observational study provides valuable insights into the clinical course, disability progression, and treatment response in pediatric patients with acquired demyelinating syndromes—specifically MS, MOGAD, and NMOSD. Our findings underscore the heterogeneity of these disorders in terms of onset characteristics, disease trajectory, and therapeutic outcomes.

The diagnostic distribution in our cohort—MS (33.3%), MOGAD (36.6%), NMOSD (6.6%), ADEM (10.0%), and other demyelinating disorders (13.3%)—shows a slightly higher proportion of MOGAD compared to MS. This may partially reflect referral bias or increased awareness and antibody testing in recent years and contrasts with the older literature identifying MS as the most common chronic ADS in children and adolescents, particularly during puberty [8,9]. The mean age at onset in our MS group (13.2 years) aligns with previous pediatric studies indicating a peak around adolescence, while MOGAD and NMOSD patients were notably younger at onset (mean age 7.4 and 6.2 years, respectively), consistent with known epidemiology [10,11].

Although the overall sex distribution in our cohort was balanced (50% male, 50% female), previous studies have consistently reported a strong female predominance in AQP4-IgG-positive NMOSD and a modest female bias in pediatric MS [9,12].

At disease onset, key topographical differences were noted. Motor disturbances and altered reflexes were the most frequent symptoms (66.7% each), followed by sensory/autonomic symptoms (50.0%) and visual disturbances (46.7%). Notably, optic neuritis predominated in MOGAD, consistent with the recognized predilection of MOG-IgG disease for bilateral optic nerve involvement, while all NMOSD patients exhibited transverse myelitis, compatible with the hallmark of longitudinally extensive transverse myelitis (LETM) [8,12].

MRI at onset showed ≥3 lesions in 76.7% of patients, most frequently in a diffuse (46.7%) or circumscribed (40.0%) pattern, with 36.7% presenting with concurrent supratentorial, infratentorial, and spinal lesions. Spinal cord involvement—either isolated or in combination with supratentorial and/or infratentorial lesions—was identified in 66.7% of patients. These early MRI findings have been shown to have known prognostic implications and were confirmed as significant predictors of EDSS and neuroradiological outcomes at later time points, with more extensive and diffuse lesions associated with less favorable outcomes. Evoked potentials, performed in 40% of the cohort, were abnormal in 20% of cases and significantly predicted disability at 1 and 12 months, indicating a negative prognostic value consistent with early subclinical dissemination. Neurocognitive assessments were found to be significantly associated with clinical evolution at 3 years, with worse cognitive performance correlating with more unfavorable clinical outcomes.

Over time, outcome trajectories diverged significantly across diagnoses. MS showed the most unfavorable progression in terms of disability: although initial improvement occurred during the first 6 months, EDSS progressively increased from the first year, consistent with cumulative disability in a chronic disease. MOGAD, in contrast, displayed a sharply favorable course, with rapid and sustained EDSS improvement and low long-term scores, indicating a positive prognosis and supporting a monophasic or fully remitting phenotype in most cases. NMOSD showed high disability at onset with limited recovery and variable stability.

The data emerging from this study are consistent with the existing literature, highlighting a more favorable disability profile in MOGAD compared to other demyelinating diseases [13,14].

In our cohort, all ADEM patients had a monophasic disease course, with no clinical relapses or new radiological lesions during follow-up.

Although ADEM is typically monophasic, recurrent and multiphasic variants have been described, characterized by new clinical episodes and radiological lesions involving different CNS regions after a defined steroid-free interval. According to Pereira et al. (2021) [15].

MRI follow-up may reveal complete resolution, partial sequelae, or new lesions suggestive of alternative diagnoses such as MS or MOGAD. None of our ADEM patients developed such features over time.

Predictors of outcome varied by time point and domain. For EDSS, early predictors included the use of DMTs (associated with more favorable outcomes) and abnormal evoked potentials (associated with worse outcomes), while later EDSS outcomes were influenced by lesion type (diffuse lesions correlated with worse prognosis), CSF profile (abnormal patterns were associated with higher disability), antibody status (positive AQP4 antibodies were associated with poorer prognosis, whereas MOG positivity correlated with better outcomes), and DMT characteristics (more targeted or early treatment strategies were linked to improved outcomes). Radiological outcomes were influenced by age at onset (younger age tended to correlate with better imaging outcomes), DMT (generally favorable), lesion characteristics (diffuse or multifocal lesions correlated with worse prognosis), and sex (with female sex associated with more severe neuroradiological progression at later timepoints). Clinical outcomes were more variable, with early predictors including sex (female sex linked to worse clinical outcomes in some cases), lesion location (brainstem and spinal lesions correlated with poorer outcomes), antibody status (MOG positivity favorable, AQP4 positivity unfavorable), diagnosis (MOGAD more favorable, MS and NMOSD less so), and DMT (generally associated with improved outcomes), but fewer consistent associations were observed beyond the first year.

Regarding treatment, acute attack therapies were widely used, with IVMP being the most common, either alone or in combination with IVIG and/or tapering steroids. Three patients (10%) recovered without treatment. DMTs were initiated in 11 patients, including Natalizumab (27.3%), Fingolimod (27.3%), and Rituximab (45.5%), the latter being used exclusively in patients with NMOSD in our cohort. Although Rituximab is frequently used in MOGAD in clinical practice, no MOGAD patients in our sample were treated with Rituximab. Its widespread use, particularly in NMOSD, reflects current clinical experience and is supported by retrospective studies demonstrating reduced relapse rates and EDSS progression, despite the lack of randomized pediatric trials [16]. While disease-modifying therapies (DMTs) are formally approved and widely used in pediatric multiple sclerosis, their off-label application in other demyelinating conditions—such as NMOSD and MOGAD—has been increasingly adopted in clinical practice, particularly in relapsing or high-risk cases. This broader use of DMTs beyond MS is also reflected in our cohort, where Rituximab was used in NMOSD patients as part of long-term immunosuppressive management.

These findings highlight the importance of early, individualized therapeutic strategies. In MS, early DMT may alter the trajectory of accumulating disability, while in NMOSD, early immunosuppression and relapse prevention are critical for long-term stability. Serial antibody testing, structured neurocognitive assessment, and neuroimaging follow-up should be integrated into routine care to identify children at risk of poor outcomes.

Limitations include the retrospective, single-center design and the small number of NMOSD cases, which limit statistical power and generalizability. The use of clinical judgment in ordering advanced tests (e.g., EEG, neurocognitive assessment) may have introduced selection bias. Moreover, some predictors were analyzed only in subsets of patients, necessitating cautious interpretation. Additionally, although spinal cord involvement was systematically coded based on topographical data, a detailed morphological analysis of spinal lesions (e.g., axial distribution, longitudinal extent, contrast enhancement) was not performed. This represents an intrinsic limitation of the retrospective design and underscores the importance of dedicated protocols in future prospective studies.

## 5. Future Directions

Several avenues for future research emerge from our findings. First, larger multicenter studies with standardized protocols are necessary to confirm the predictive value of early clinical, radiological, and immunological features, especially in rarer entities like NMOSD and relapsing MOGAD. Future studies should ensure systematic performance of EEG, evoked potentials, neurocognitive testing, and serial antibody titration to improve risk stratification.

Second, longitudinal integration of quantitative imaging markers and fluid biomarkers, such as serum or CSF neurofilament light chain (NfL), could enhance early identification of children at risk of subclinical progression or future relapses [17].

Third, randomized controlled trials are needed to guide the use of long-term immunotherapies—particularly Rituximab—in pediatric MOGAD and NMOSD, where treatment decisions are currently extrapolated from adult data or based on retrospective evidence.

Finally, the development of personalized treatment algorithms, incorporating clinical phenotype, antibody status, neuroimaging features, and functional testing, may optimize outcomes and quality of life in children with acquired demyelinating syndromes.

## 6. Conclusions

This study provides important insights into the clinical heterogeneity and treatment response in pediatric patients with acquired demyelinating syndromes, including MS, MOGAD, and NMOSD. The findings confirm the diverse presentations and disease courses of these disorders and underscore the need for timely and accurate diagnosis to guide effective therapeutic management.

MOGAD typically exhibited a more favorable trajectory and prompt treatment response, particularly in monophasic forms, supporting the avoidance of overly aggressive long-term immunosuppression in selected cases. In contrast, pediatric MS was associated with progressive disability, emphasizing the importance of early and intensive initiation of disease-modifying therapies to alter its course [18,19].

The persistence of clinical activity and limited recovery observed in NMOSD, especially in AQP4-IgG-positive patients, highlights the need for sustained immunosuppressive strategies and close long-term follow-up [8,20,21,22,23].

A multidimensional follow-up approach—incorporating clinical, neuroradiological, immunological, and cognitive parameters—proved essential for predicting outcomes and tailoring treatment over time. This study further supports the utility of a comprehensive diagnostic assessment integrating neuroimaging, antibody profiling, CSF analysis, evoked potentials, and neurocognitive evaluation to stratify risk and personalize management [24,25].

Given the rarity of and variability in these conditions, larger prospective multicenter studies with standardized protocols are needed to validate and expand upon these preliminary findings and better elucidate the underlying pathogenic mechanisms [26].

The study supports the utility of comprehensive diagnostic workups, which integrate neuroimaging, CSF analysis, antibody profiling, evoked potentials, and neurocognitive assessment, to stratify risk and guide personalized treatment plans [12,27].

However, limitations such as small sample size, retrospective design, and heterogeneous availability of diagnostic tests emphasize the need for larger, multicenter prospective studies with standardized protocols to confirm and expand these preliminary observations [28].

## Figures and Tables

**Table 1:** Demographic, clinical, and diagnostic characteristics of the pediatric cohort with acquired demyelinating syndromes.

*Variable*	Sample Size (n = 30)
**Male gender**, n (%)	15 (50)
**Age at the onset <11 years,** n (%)	15 (50)
**Positive familiarity for neurological disorders,** (%)	13
**Positive familiarity for autoimmune disease,** (%)	20
**EBV,** n (%)	16 (53)
**CSF hyperproteinorrachia,** n (%)	10 (33)
**Diagnosis,** (%)	
MS	33
MOGAD	36.6
NMO	6.6
ADEM	10
Other	13.3
**Symptoms at onset,** n (%)	
Motor disturbances and altered reflex	20 (66.7)
Sensory disturbances	15 (50)
Visual impairments	14 (46.7)
Altered states of consciousness	11 (36.7)
Cranial nerve involvement	3 (10)
Coordination and balance issues	11 (36.7)
Seizure	1 (3.3)
**Acute attack therapy,** n (%)	
Yes	27 (90)
**DMT therapy,** n (%)	
Yes	11 (36)
**Type of DMD therapy,** (%)	
Natalizumab	27.3
Fingolimod	27.3
Rituximab	45.5

## Data Availability

The datasets generated and/or analyzed during the current study are available from the corresponding author upon reasonable request due to privacy.
